# Robust Statistical Frontalization of Human and Animal Faces

**DOI:** 10.1007/s11263-016-0920-7

**Published:** 2016-07-20

**Authors:** Christos Sagonas, Yannis Panagakis, Stefanos Zafeiriou, Maja Pantic

**Affiliations:** 1 Department of Computing, Imperial College London, 180 Queens Gate, London, SW7 2AZ UK; 2 Faculty of Electrical Engineering, Mathematics and Computer Science, University of Twente, Enschede, The Netherlands

**Keywords:** Pose normalization, Landmark localization, Face recognition, Low rank, Sparsity

## Abstract

The unconstrained acquisition of facial data in real-world conditions may result in face images with significant pose variations, illumination changes, and occlusions, affecting the performance of facial landmark localization and recognition methods. In this paper, a novel method, robust to pose, illumination variations, and occlusions is proposed for joint face frontalization and landmark localization. Unlike the state-of-the-art methods for landmark localization and pose correction, where large amount of manually annotated images or 3D facial models are required, the proposed method relies on a small set of frontal images only. By observing that the frontal facial image of both humans and animals, is the one having the minimum rank of all different poses, a model which is able to jointly recover the frontalized version of the face as well as the facial landmarks is devised. To this end, a suitable optimization problem is solved, concerning minimization of the nuclear norm (convex surrogate of the rank function) and the matrix $\ell _1$ norm accounting for occlusions. The proposed method is assessed in frontal view reconstruction of human and animal faces, landmark localization, pose-invariant face recognition, face verification in unconstrained conditions, and video inpainting by conducting experiment on 9 databases. The experimental results demonstrate the effectiveness of the proposed method in comparison to the state-of-the-art methods for the target problems.

## Introduction

Face frontalization refers to the recovery of the frontal view of faces from images captured in unconstrained conditions. Accurate face frontalization is a cornerstone for many face analysis problems. For example, recently, it has been shown that well-designed face frontalization can help in achieving state-of-the-art performance in face recognition in unconstrained conditions (Taigman et al. 2014; Hassner et al. 2015).^1^

**Fig. 1:**
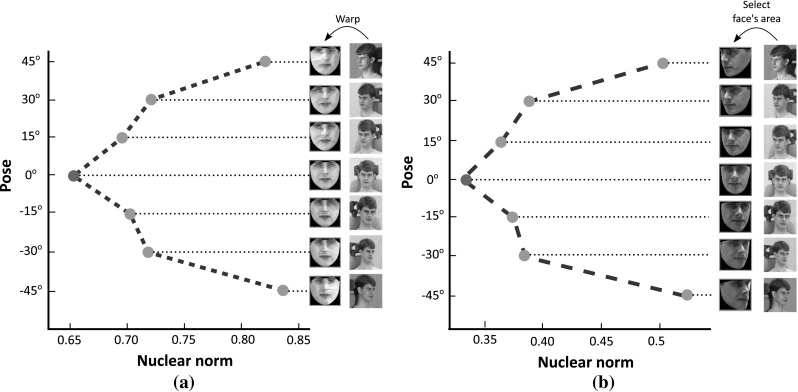
The average value of nuclear norm computed based on **a** warped into a common frame and **b** cropped ‘Neutral’ images of twenty subjects from Multi-PIE database under poses $-45^\circ$ to $45^\circ$. The initial images, warped, and cropped ones are also depicted

An essential step towards face frontalization is facial landmark localization. State-of-the-art landmark localization methods (Tzimiropoulos et al. 2013; Saragih et al. 2011; Asthana et al. 2013; Xiong and De la Torre 2013; Ren et al. 2014; Kazemi and Sullivan 2014) model the problem discriminatively by capitalizing on the availability of annotated data (in terms of facial landmarks) (Sagonas et al. 2013b, a, 2016). Unfortunately, the annotation of facial landmarks is laborious, expensive, and time consuming process. This is even more the case for faces that are not in frontal pose.^2^

In many cases, even accurate 2D landmark localization is not enough for successful face frontalization. That is, the frontalization step often requires both landmark localization and pose correction by usually resorting to 3D face models (Taigman et al. 2014; Yi et al. 2013; Sun et al. 2014; Hassner et al. 2015). In general, 3D model-based methods employ a 3D dense surface model in order to compute the 3D face shape, as well as the pose of the face depicted in an image. Then, the recovered shape is used to synthesize the frontal view of the face. However, such methods cannot be widely applied since they require: (a) a method for accurate landmark localization in various poses, (b) fitting learned 3D generic model of face, which is expensive to built, and (c) a robust image warping algorithm for frontal view image reconstruction (Taigman et al. 2014). As an alternative to this process, the authors of (Hassner et al. 2015) propose to avoid 3D face model fitting by employing a single 3D reference mesh.

In contrast to the 3D-model based methods, the patch-based methods approximate 3-D pose transformations as a set of linear transformations of 2D image patches. For instance, the Lucas-Kanade algorithm is employed to align patches of non-frontal faces to the corresponding one in frontal facial images (Ashraf et al. 2008). In (Chai et al. 2007; Li et al. 2012a), face frontalization is obtained via locally linear regression of patches, while (Ho and Chellappa 2013) employs a Markov Random Field (MRF). The main drawback of the latter is that for each non-frontal image, an exhaustive batch-based alignment algorithm is applied (trained on frontal patches), resulting in a time consuming procedure. In addition, the semantic correspondence between the non-frontal (test) and frontal (train) patches can be lost when significant pose variations occur. It is worth mentioning that, the patch-based methods are not able to handle adequately local non-linear deformations, which appear within the patch.

Furthermore, pose normalization is beneficial for fine-grained categorization (i.e., subcategory recognition) in different classes of objects e.g., cats and dogs (Parkhi et al. 2012; Liu et al. 2012), flowers(Angelova et al. 2013; Nilsback and Zisserman 2006), birds (Deng et al. 2013; Gavves et al. 2013), and cars (Krause et al. 2013; Lin et al. 2014). Current state-of-the-art methods (Branson et al. 2014; Zhang et al. 2014) rely upon the use of 2D annotations in order to build convolutional neural networks for pose-normalized representations of objects. However, these methods can not be applied widely in different objects since object-specific annotations are required. Clearly such a procedure is cost-prohibitive. On the other hand, the use of 3D models is limited to 3D CAD car models (Lin et al. 2014), while 3D models of other arbitrary objects such as cats, dogs, and rabbits are either limited or do not exist at all and in general is expensive to acquire.

**Fig. 2:**
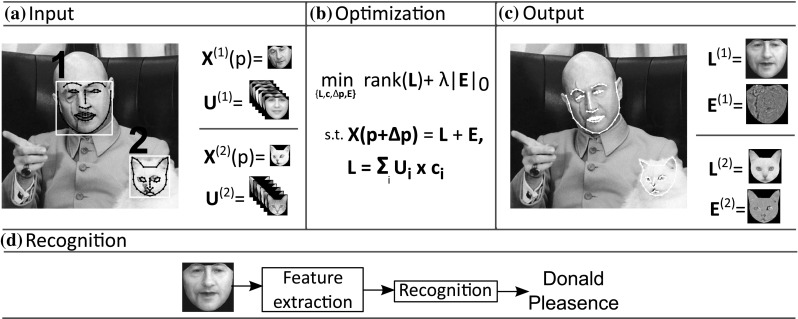
Flowchart of the proposed method: **a** Given an input image, the results from a detector, and a statistical model $\mathbf{U}$, built on frontal images only, **b** a constrained low-rank minimization problem is solved. **c** Face alignment and frontal view reconstruction are performed simultaneously. Finally, **d** face recognition is performed using the frontalized image

In this paper, we propose a unified method for joint face frontalization (pose correction) and landmark localization, using a small set of frontal images only. The key motivational observation is that for facial images lying in a linear symmetric space, the rank of a frontal facial image is much smaller than the rank of facial images in other poses. To demonstrate the above observation, ‘Neutral’ images of twenty subjects from Multi-PIE database (Gross et al. 2010) under poses $-45^\circ$ to $45^\circ$ were warped into a reference frontal-pose frame and the nuclear norm (convex surrogate of the rank) of each shape-free texture was computed. In Fig. 1a the average value of the nuclear norm for different poses is reported. Clearly, the frontal pose has the smallest nuclear norm value compared to the corresponding values computed for other poses. Furthermore, the above observation was verified in case where the faces are not warped into a reference frame. To this end, the images used in the previous experiment were aligned based on the outer corner of their eyes. Then, using the landmark points of each aligned face we found the corresponding face convex hull and set equal to zero all the pixels that do not belong to this. Subsequently, the same bounding box was used in order to crop the face area in each image. In Fig. 1b the average value of the nuclear norm computed from the cropped images for different poses is reported. As it can been observed the frontal pose has the smallest nuclear norm value compared to the corresponding values computed for other poses. However, severe deviations from the above linear facial model occur in the presence of pose, occlusions, expressions, and illumination changes.

The proposed method: (a) approximately removes deformations due to pose and expressions by exploiting a motion model, (b) models occlusion/specular highlights and warping errors as noise (that is sparser than the actual signal), and (c) handles illumination variations by employing *in-the-wild* frontal facial images by solving a suitable optimization problem, involving the minimization of the nuclear norm and the matrix $\ell _1$ norm. The flowchart of the proposed method (coined as RSF–*Robust Statistical Face Frontalization*) is depicted in Fig. 2.

The most closely related work to the RSF is the Transform Invariant Low-rank Textures (TILT) (Zhang et al. 2012), where texture rectification is obtained by applying a global affine transformation onto a low-rank term, modelling the texture. By blindly imposing low-rank constraints without regularization, for non-rigid alignment opposite effects may occur. As recently demonstrated (Cheng et al. 2013a; Sagonas et al. 2014; Cheng et al. 2013b), non-rigid deformable models cannot be straightforward combined with optimization problems (Peng et al. 2012) that involve low-rank terms without a proper regularization. To overcome the aforementioned problems, a model of frontal images is employed in this work.

The contributions of the paper are summarized as follows:

- A novel method, i.e., the RSF,^3^ for joint landmark localization and face frontalization is proposed to adequately model pose, occlusions, expressions, and illumination variations using a statistical model of frontal images, low-rank, and sparsity. Furthermore, the RSF is extended to F-RSF for handling multi-channel image representations (i.e., features such as SIFT, IGO, HoG etc) and to RSF-V for joint frontalization and alignment in a batch of images or videos.
- The performance of RSF is assessed by conducting extensive experiments using human faces, cat faces, and face sketches from 9 databases. The effectiveness of the RSF-V is demonstrated in video-based face verification and video inpainting.
- We demonstrate, for the first time, that it is possible to **improve** the **state-of-the-art** results in generic landmark localization, pose-invariant face recognition, and unconstrained image and video face verification tasks by using a model of **frontal images only**. This finding is surprising since it implies that when phenomena are properly modelled, simple **statistical linear models suffice**.

The proposed methodology can aid the design of applications in two ways: (a) in case there exist many available annotated data, it can largely boost the performance of learning-based recognition methods (as frontalisation achieves in (Taigman et al. 2014)) and (b) it can aid in achieving state-of-the-art (or competitive) results in challenging settings where there is still of lack of data [e.g. the restricted protocols of LFW (Huang et al. 2007)] or in cases in which annotated data are expensive to acquire (e.g., landmark localisation).

The remainder of the paper is organized as follows. In Sect. 2 basic notations and definitions are introduced. The RSF, F-RSF, and RSF-V methods are detailed in Sects. 3 and 4, respectively. In Sect. 5 the experimental results are presented. Section 6 concludes the paper.

## Notations and Preliminaries

Throughout the paper, scalars are denoted by lower-case letters, vectors (matrices) are denoted by lower-case (upper-case) boldface letters i.e., $\mathbf{x}$, ($\mathbf{X}$). $\mathbf{I}$ denotes the identity matrix. The *i*th column of $\mathbf{X}$ is denoted by $\mathbf{x}_{i}$. A vector $\mathbf{x} \in \mathbb {R}^{m \cdot n}$ (matrix $\mathbf{X} \in \mathbb {R}^{m \times n}$) is reshaped into a matrix (vector) via the reshape operator : $\mathcal {R}_{m \times n}(\mathbf{x}) = \mathbf{X} \in \mathbb {R}^{m \times n}$, $\big (\text{ vec }(\mathbf{X}) = \mathbf{x} \in \mathbb {R}^{m\cdot n \times 1}\big )$.

The ${{\mathrm{rank}}}(\mathbf{X})$ is the rank of a matrix $\mathbf{X}$ (i.e., the maximum number of linearly independent rows or columns in $\mathbf{X}$). The $\ell _1$ and the $\ell _2$ norms of $\mathbf{x}$ are defined as $\left\| \mathbf{x} \right\| _1 = \sum _i \vert x_i\vert$ and $\left\| \mathbf{x} \right\| _2 = \sqrt{\sum _i x_i^2}$, respectively. The matrix $\ell _1$ norm is defined as $\left\| \mathbf{X} \right\| _1 = \sum _i\sum _j \vert x_{ij}\vert$, where $\vert \cdot \vert$ denotes the absolute value operator. The Frobenius norm is defined as $\left\| \mathbf{X} \right\| _F = \sqrt{\sum _i\sum _j x_{ij}^2}$, and the nuclear norm of $\mathbf{X}$ (i.e., the sum of singular values of a matrix) is denoted by $\left\| \mathbf{X} \right\| _*$. $\mathbf{X}^T$ is the transpose of $\mathbf{X}$. If $\mathbf{X}$ is a square matrix, $\mathbf{X}^{-1}$ is its inverse, provided that the inverse matrix exists. The *i*-th vector of the standard basis in $\mathbb {R}^{m\cdot n}$ is denoted as $\mathbf{q}_{m\cdot n}^{(i)}, i = 1,\dots , m \cdot n$.

A shape instance consisting of *N* landmark points is denoted as $\mathbf{s}$ = $[x^{(1)},y^{(1)},\ldots ,x^{(N)},y^{(N)}]$. A small set of shape instances $\{\mathbf{s}_i\}$ is used to learn a point distribution model (PDM). First, all the shapes are put into correspondence by removing the global similarity transforms via Generalized Procrustes Analysis. Then, a principal component analysis (PCA) is applied on the aligned shapes, resulting in a number of $N_S$ eigen-shapes $\mathbf{U}_{S}$ and the mean shape $\bar{\mathbf{s}}$. Given a PDM $\mathcal {S}=\{\bar{\mathbf{s}},\mathbf{U}_S \in \mathbb {R}^{2N\times N_S}\}$ a new instance is generated as $\mathbf{s} = \bar{\mathbf{s}} + \mathbf{U}_S\mathbf{p}$, where $\mathbf{p}$ is the $N_S \times 1$ vector of shape parameters. The warp function $\mathbf{x}(\mathcal {W}({\mathbf {z}};\mathbf{p}))$ $\big (\mathbf{X}(\mathcal {W}({\mathbf {z}}; \mathbf{p}))\big )$ denotes the warping of each 2D point ${\mathbf {z}}=[x,y]$ within a shape instance to its corresponding location in a reference frame. To simplify the notation $\mathbf{x}(\mathbf{p}) \big (\mathbf{X}(\mathbf{p})\big )$ will be used throughout the paper instead of $\mathbf{x}(\mathcal {W}({\mathbf {z}},\mathbf{p}))$ $\big (\mathbf{X}(\mathcal {W}({\mathbf {z}},\mathbf{p}))\big )$. Finally, the reference frame is defined when $\mathbf{p}={\mathbf {0}}$, such that $\mathbf{x}(\mathbf{p})=\mathbf{x}\big (\mathbf{X}(\mathbf{p})=\mathbf{X}\big )$.

## Robust Face Frontalization

### Problem Statement

Let $\mathbf{X}\in \mathbb {R}^{h\times r}$ be an image depicting a non-frontal view of a face and $\mathbf{s}\in \mathbb {R}^{2N\times 1}$ an initial estimation of *N* landmark points, describing the shape. To create a shape-free texture, the input image is warped into a frontal-pose reference frame by employing a warp function $\mathcal {W}(\cdot )$. In many cases the warped image $\mathbf{X}(\mathbf{p})\in \mathbb {R}^{m\times n}$ can be corrupted by sparse errors of large magnitude. Such sparse errors indicate that only a small fraction of the image pixels may be corrupted by non-Gaussian noise and occlusions. In this paper, the goal is to recover the clean frontal view (i.e., a low-rank image ${\mathbf {L}} \in \mathbb {R}^{m \times n}$) of the $\mathbf{X}(\mathbf{p})$ such that: $\mathbf{X}(\mathbf{p}) = {\mathbf {L}}+\mathbf{E}$, where $\mathbf{E} \in \mathbb {R}^{m \times n}$ is a sparse error matrix, accounting for gross errors. This formulation leads to the following optimization problem:

1 $$\begin{aligned} \underset{\{{\mathbf {L}},\mathbf{p},\mathbf{E}\}}{\mathop {\hbox {argmin }}\limits } {{\mathrm{rank}}}({\mathbf {L}}) + \lambda \left\| \mathbf{E} \right\| _0, \quad \text {s.t.} \quad \mathbf{X}(\mathbf{p}) ={\mathbf {L}} + \mathbf{E}. \end{aligned}$$

**Fig. 3:**
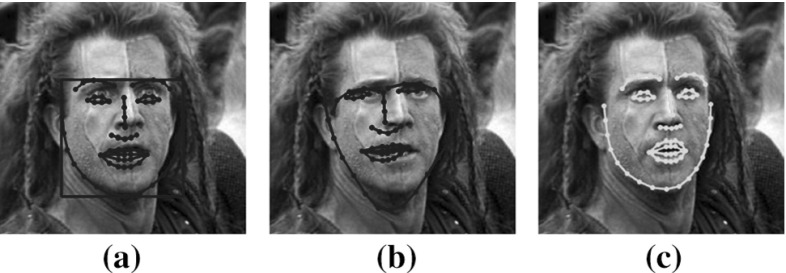
**a** Input image and initial position of the landmarks. Results obtained by the **b** TILT and **c** RSF

In (Zhang et al. 2012), TILT transforms the above  non-convex problem into convex (Candès et al. 2011) and subsequently solves efficiently the relaxed problem in an alternating fashion (Bertsekas 1982). However, by minimizing the  non-regularized rank of the image ensemble, tends to unnaturally deform the subject’s facial appearance resulting in false face alignment (Sagonas et al. 2014; Cheng et al. 2013a). Figure 3a, b show the initial position of the landmarks used as initialization (Zhu and Ramanan 2012) and the corresponding result obtained by the TILT, respectively. As it can be seen the result is very poor which is expected due to the lack of regularization in the rank constraint.

In order to solve the above problem and ensure that unnatural faces will not be created, a statistical model built from frontal images is utilized. In particular, based on the observation that the frontal view of a face is in a low-rank subspace (please refer to Fig. 1), it can be expressed as a linear combination of a small number of precomputed orthonormal bases (i.e.  $\mathbf{U} =[\mathbf{u}_1 | \mathbf{u}_2 | \cdots | \mathbf{u}_k] \in \mathbb {R}^{m\cdot n \times k}$,  $\mathbf{U}^T\mathbf{U} = \mathbf{I}$) that span a generic (clean) frontal view subspace, that is ${\mathbf {L}} = \sum _{i=1}^k \mathcal {R}_{m \times n}(\mathbf{u}_i) c_i$. Therefore, the deformed corrupted input image is written as: $\mathbf{X}(\mathbf{p})={\mathbf {L}}+\mathbf{E}=\sum _{i=1}^k \mathcal {R}_{m \times n}(\mathbf{u}_i) c_i + \mathbf{E}$.

To match the specifications of the frontal image and the sparse error one can find the low-rank frontal image, the linear combination coefficients, the increments of warp parameters, and the error matrix by solving the following optimization problem:2where $\lambda$ is a positive weighting parameter that balances the rank of ${\mathbf {L}}$ and the sparsity of the $\mathbf{E}$. Problem (2) is difficult to be solved since: (a) both rank function and $\ell _0$-norm are non-convex, discrete valued functions, minimization of which is NP-hard (Natarajan 1995; Vandenberghe and Boyd 1996), and (b) the constraint $\mathbf{X}(\mathbf{p}) = {\mathbf {L}} + \mathbf{E}$ is non-linear. To alleviate this problem, the nuclear- and the $\ell _1$- norms are adopted as convex surrogates to rank function and $\ell _0$- norm (Fazel 2002; Donoho 2006). To address the non-linearity of the above mentioned equality constraint, a first order Taylor linear approximation is applied on the vectorized form of the constrained: $\mathbf{x}(\mathbf{p}+{\varDelta }\mathbf{p}) \approx \mathbf{x}(\mathbf{p}) + \mathbf{J}(\mathbf{p}){\varDelta }\mathbf{p}$, where $\text{ vec }(\mathbf{X}(\mathbf{p})) = \text{ vec }({\mathbf {L}} + \mathbf{E}) = \mathbf{U}\mathbf{c} + \mathbf{e}= \mathbf{x}(\mathbf{p})$ and $\mathbf{J}(\mathbf{p})=\nabla \mathbf{x}(\mathbf{p}) \frac{\partial \mathcal {W}}{\partial \mathbf{p}}$ is the Jacobian matrix with the steepest descent images as its columns. Consequently, the RSF solves the following optimization problem:

3 $$\begin{aligned} \begin{aligned}&\underset{\{{\mathbf {L}},\mathbf{c},{\varDelta }\mathbf{p},\mathbf{e}\}}{\mathop {\hbox {argmin }}\limits } \left\| {\mathbf {L}} \right\| _* + \lambda \left\| \mathbf{e} \right\| _1 \\&\text {s.t.} \left\{ \begin{array}{ll} H^{(1)}({\varDelta }\mathbf{p}, \mathbf{c}, \mathbf{e})=\mathbf{x}(\mathbf{p}) + \mathbf{J}(\mathbf{p}){\varDelta }\mathbf{p} - \mathbf{U}\mathbf{c} -\mathbf{e}={\mathbf {0}}\\ H^{(2)}({\mathbf {L}},\mathbf{c}) = {\mathbf {L}} - \sum _{i=1}^k \mathcal {R}_{m \times n}(\mathbf{u}_i) c_i={\mathbf {0}}. \end{array}\right. \end{aligned} \end{aligned}$$

### Alternating-Direction Based-Method Algorithm

To solve (3), the *augmented* Lagrangian (Bertsekas 1982) is introduced:

4 $$\begin{aligned}&\mathcal {L}({\mathbf {L}},\mathbf{c},{\varDelta }\mathbf{p}, \mathbf{e},\mathcal {M}) = \left\| {\mathbf {L}} \right\| _* + \lambda \left\| \mathbf{e} \right\| _1 + \mathbf{a}^TH^{(1)}({\varDelta }\mathbf{p}, \mathbf{c}, \mathbf{e})\nonumber \\&\quad +\, {\mathrm {{tr}}}\left( {\mathbf {B}}^TH^{(2)}({\mathbf {L}},\mathbf{c})\right) + \frac{\mu }{2}\left\| H^{(1)}({\varDelta }\mathbf{p}, \mathbf{c}, \mathbf{e}) \right\| _2^2 \nonumber \\&\quad +\,\frac{\mu }{2}\left\| H^{(2)}({\mathbf {L}},\mathbf{c}) \right\| _F^2 , \end{aligned}$$

where $\mathcal {M} = \{\mathbf{a} \in \mathbb {R}^{m\cdot n} , {\mathbf {B}} \in \mathbb {R}^{m \times n}\}$ are the Lagrange multipliers for the equality constraints in (3) and $\mu > 0$ is a penalty parameter. Equivalently, (4) can be rewritten as follows:

5 $$\begin{aligned}&\mathcal {L}({\mathbf {L}},\mathbf{c},{\varDelta }\mathbf{p},\mathbf{e},\mathcal {M}) = \left\| {\mathbf {L}} \right\| _* + \lambda \left\| \mathbf{e} \right\| _1 \nonumber \\&\quad +\, \frac{\mu }{2}\left\| H^{(1)}({\varDelta }\mathbf{p}, \mathbf{c}, \mathbf{e}) + \frac{\mathbf{a}}{\mu } \right\| _2^2 + \frac{\mu }{2}\left\| H^{(2)}({\mathbf {L}},\mathbf{c}) + \frac{{\mathbf {B}}}{\mu } \right\| _F^2 \nonumber \\&\quad -\,\frac{1}{2\mu }\left( \left\| \mathbf{a} \right\| _2^2 +\left\| {\mathbf {B}} \right\| _F^2 \right) . \end{aligned}$$

By employing the alternating directions method of multipliers (ADMM) (Bertsekas 1982),  (3) is solved by minimizing  (4) with respect to each variable in an alternating fashion. Finally, the Lagrange multipliers are updated at each iteration.

Let *t* be the iteration index. For notation convenience we will write $\mathcal {L}({\mathbf {L}}_{[t]})$ instead of $\mathcal {L}({\mathbf {L}}_{[t]},\mathbf{c}_{[t]},{\varDelta }\mathbf{p}_{[t]},\mathbf{e}_{[t]},\mathcal {M}_{[t]})$ when all the variables except ${\mathbf {L}}_{[t]}$ are kept fixed. Accordingly, given ${\mathbf {L}}_{[t]},\mathbf{c}_{[t]},{\varDelta }\mathbf{p}_{[t]},\mathbf{e}_{[t]},\mathcal {M}_{[t]}$ and $\mu _{[t]}$, the iterations reads as follows:

6 $$\begin{aligned} {\mathbf {L}}_{[t+1]} =&\mathop {\hbox {argmin }}\limits _{{\mathbf {L}}_{[t]}} \mathcal {L}({\mathbf {L}}_{[t]}), \end{aligned}$$

7 $$\begin{aligned} \mathbf{c}_{[t+1]} =&\mathop {\hbox {argmin }}\limits _{\mathbf{c}_{[t]}} \mathcal {L}(\mathbf{c}_{[t]}), \end{aligned}$$

8 $$\begin{aligned} {\varDelta }\mathbf{p}_{[t+1]} =&\mathop {\hbox {argmin }}\limits _{{\varDelta }\mathbf{p}_{[t]}} \mathcal {L}({\varDelta }\mathbf{p}_{[t]}), \end{aligned}$$

9 $$\begin{aligned} \mathbf{e}_{[t+1]} =&\mathop {\hbox {argmin }}\limits _{\mathbf{e}_{[t]}} \mathcal {L}(\mathbf{e}_{[t]}). \end{aligned}$$

**Step 1**: Update $${\mathbf {L}}$$:

10 $$\begin{aligned} {\mathbf {L}}_{[t+1]} =\mathop {\hbox {argmin }}\limits _{{\mathbf {L}}_{[t]}} \left\| {\mathbf {L}}_{[t]} \right\| _* + \frac{\mu }{2}\left\| H^{(2)}({\mathbf {L}}_{[t]},\mathbf{c}_{[t]}) + \frac{{\mathbf {B}}_{[t]}}{\mu _{[t]}} \right\| _F^2 . \end{aligned}$$

The nuclear norm regularized least squared problem (10) has the following closed-form solution:

11 $$\begin{aligned} {\mathbf {L}}_{[t+1]} = \mathcal {D}_{\frac{1}{\mu _{[t]}}}\left[ \sum _{i=1}^k \mathcal {R}(\mathbf{u}_i)c_{i,[t]} - \frac{{\mathbf {B_{[t]}}}}{\mu _{[t]}} \right] . \end{aligned}$$

The singular value thresholding (SVT) operator is defined for any matrix $\mathbf{Q}$ with $\mathbf{Q}=\mathbf{U} {{\mathbf {\Sigma }}} \mathbf{V}^T$ as $\mathcal {D}_{\tau }[\mathbf{Q}] = \mathbf{U}{{\mathbf {\mathcal {S}}}}_{\tau }\mathbf{V}^T$ (Cai et al. 2010), with ${{\mathbf {\mathcal {S}}}}_{\tau }[\sigma ] =$sgn$(\sigma )\max (|\sigma |-\tau ,0)$ being the (element-wise) shrinkage operator (Candès et al. 2011).

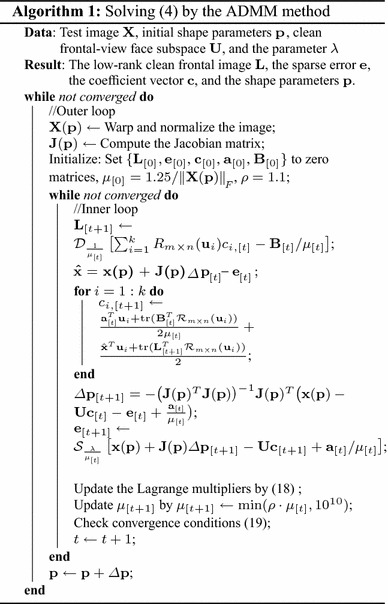



**Step 2**: Update $$\mathbf{c}$$:

12 $$\begin{aligned} \mathbf{c}_{[t+1]}&= \mathop {\hbox {argmin }}\limits _{\mathbf{c}_{[t]}} \text { } \frac{\mu _{[t]}}{2}\left( \big \Vert H^{(1)}({\varDelta }\mathbf{p}_{[t]}, \mathbf{c}_{[t]}, \mathbf{e}_{[t]})+\frac{\mathbf{a}_{[t]}}{\mu _{[t]}}\big \Vert _2^2\right. \nonumber \\&\quad \left. +\, \big \Vert H^{(2)}({\mathbf {L}}_{[t+1]},\mathbf{c}_{[t]}) + \frac{{\mathbf {B}}_{[t]}}{\mu _{[t]}}\big \Vert _F^2 \right) . \end{aligned}$$

(12) is a quadratic problem which admits a closed form solution given element-wise by:

13 $$\begin{aligned} c_{i,[t+1]} = \frac{\mathbf{a}_{[t]}^T\mathbf{u}_i+{\mathrm {{tr}}}({\mathbf {B}}_{[t]}^T\mathcal {R}(\mathbf{u}_i))}{2\mu _{[t]}} + \frac{\hat{\mathbf{x}}^T\mathbf{u}_i + {\mathrm {{tr}}}({\mathbf {L}}^T_{[t+1]}\mathcal {R}(\mathbf{u}_i))}{2}, \end{aligned}$$

where $$\hat{\mathbf{x}} = \mathbf{x}(\mathbf{p}) + \mathbf{J}(\mathbf{p}){\varDelta }\mathbf{p}_{[t]} -\mathbf{e}_{[t]}$$.

**Step 3**: Update $${\varDelta }\mathbf{p}$$:

14 $$\begin{aligned} {\varDelta }\mathbf{p}_{[t+1]} = \mathop {\hbox {argmin }}\limits _{{\varDelta }\mathbf{p}_{[t]}} \text { } \frac{\mu _{[t]}}{2} \left\| H^{(1)}({\varDelta }\mathbf{p}_{[t]}, \mathbf{c}_{[t+1]}, \mathbf{e}_{[t]})+\frac{\mathbf{a}_{[t]}}{\mu _{[t]}} \right\| _2^2. \end{aligned}$$

The increment of the parameters ${\varDelta }\mathbf{p}$ is computed by solving the least square problem (14):

15 $$\begin{aligned}&{\varDelta }\mathbf{p}_{[t+1]} \nonumber \\&\quad =-\left( \mathbf{J}(\mathbf{p})^T\mathbf{J}(\mathbf{p})\right) ^{-1}\mathbf{J}(\mathbf{p})^T \left( \mathbf{x}(\mathbf{p})-\mathbf{U}\mathbf{c}_{[t+1]}-\mathbf{e}_{[t]} \!+\! \frac{\mathbf{a}_{[t]}}{\mu _{[t]}}\right) \!. \end{aligned}$$

**Step 4**: Update $$\mathbf{e}$$:

16 $$\begin{aligned} \mathbf{e}_{[t+1]}&= \mathop {\hbox {argmin }}\limits _{\mathbf{e}_{[t]}} \lambda \left\| \mathbf{e}_{[t]} \right\| _1\nonumber \\&\quad +\,\frac{\mu _{[t]}}{2} \left\| H^{(1)}({\varDelta }\mathbf{p}_{[t+1]}, \mathbf{c}_{[t+1]}, \mathbf{e}_{[t]}) +\frac{\mathbf{a}_{[t]}}{\mu _{[t]}} \right\| _2^2. \end{aligned}$$

The closed-form solution of (16) is given by applying element-wise the shrinkage operator onto: $\mathbf{x}(\mathbf{p}) + \mathbf{J}(\mathbf{p}){\varDelta }\mathbf{p} - \mathbf{U}\mathbf{c}_{[t+1]} + \mathbf{a}_{[t]}/ \mu _{[t]}$, namely:

17 $$\begin{aligned} \mathbf{e}_{[t+1]} = \mathcal {S}_{\frac{\lambda }{\mu _{[t]}}}\left[ \mathbf{x}(\mathbf{p}) + \mathbf{J}(\mathbf{p}){\varDelta }\mathbf{p}_{[t+1]} - \mathbf{U}\mathbf{c}_{[t+1]} + \frac{\mathbf{a}_{[t]}}{\mu _{[t]}} \right] . \end{aligned}$$

**Step 5**: Update Lagrange multipliers $\mathbf{a}, {\mathbf {B}}$ and $\mu$ : The Lagrange multipliers and the parameter $\mu$ are updated by:

18 $$\begin{aligned} \left\{ \begin{array}{l} \mathbf{a}_{[t+1]} = \mathbf{a}_{[t]} + \mu _{[t]} \cdot H^{(1)}({\varDelta }\mathbf{p}_{[t+1]},\mathbf{c}_{[t+1]},\mathbf{e}_{[t+1]})\\ {\mathbf {B}}_{[t+1]} = {\mathbf {B}}_{[t]} + \mu _{[t]} \cdot H^{(2)}({\mathbf {L}}_{[t+1]},\mathbf{c}_{[t+1]})\\ \mu _{[t+1]} = \min (\rho \cdot \mu _{[t]}, 10^{10}) \end{array}\right. \end{aligned}$$

*Convergence Criteria:* The inner loop of the Algorithm 1 terminates when:

19 $$\begin{aligned} \left\{ \begin{array}{l} \max \big (\left\| \mathbf{e}_{[t+1]} - \mathbf{e}_{[t]} \right\| _2/\left\| \mathbf{x}(\mathbf{p}) \right\| _2,\\ \left\| {\mathbf {L}}_{[t+1]} - {\mathbf {L}}_{[t]} \right\| _F/\left\| \mathbf{x}(\mathbf{p}) \right\| _2\big ) \le \epsilon _1\\ \max \big (\left\| H^{(1)}({\varDelta }\mathbf{p}_{[t+1]}, \mathbf{c}_{[t+1]}, \mathbf{e}_{[t+1]}) \right\| _2/\left\| \mathbf{x}(\mathbf{p}) \right\| _2,\\ \left\| H^{(2)}({\mathbf {L}}_{[t+1]},\mathbf{c}_{[t+1]}) \right\| _F/\left\| \mathbf{x}(\mathbf{p}\big ) \right\| _2) \le \epsilon _2\\ \end{array}\right. \end{aligned}$$

The Alg. 1 terminates when the change of the $\left\| {\mathbf {L}} \right\| _* + \lambda \left\| \mathbf{E} \right\| _1$ between two successive iterations is smaller than a predefined threshold $\epsilon _3$ or the maximum number of the outers’ loop iterations is reached.

**Fig. 4:**
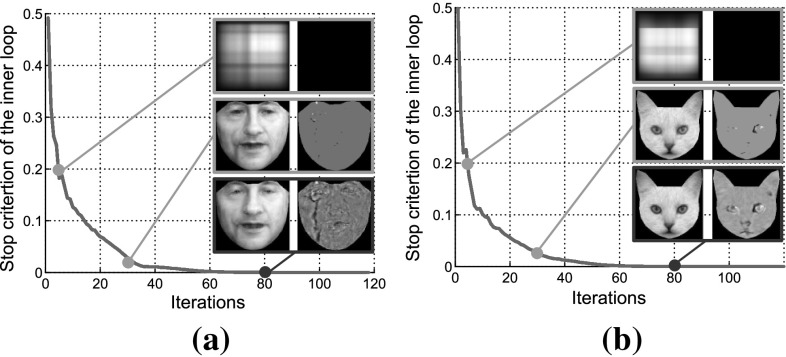
The convergence curve of the Algorithm’s 1 inner loop in case of **a** human face and **b** cat face

*Computational Complexity:* The dominant cost of each iteration of Algorithm 1 is that of the Singular Value Decomposition (SVD) algorithm involved in the computation of the SVT operator in update of ${\mathbf {L}}$ (Step 1). Consequently, the computational complexity of Algorithm 1 is $\mathcal {O}(T(min(m, n)^3 + n^2m))$, where *T* is the total number of iterations until convergence.

*Convergence:* Regarding the convergence of the Algorithm 1 there is currently no theoretical proof known for the ADMM in problems with more than two blocks of variables. However ADMM has been applied successfully in non convex optimization problems in practice (Sagonas et al. 2014; Peng et al. 2012; Panagakis et al. 2015; Georgakis et al. 2016; Papamakarios et al. 2014). In addition, the thorough experimental evaluation of the proposed method, presented in Sect. 5, indicates that the convergence of Algorithm 1 is empirically proved for data that RSF tested. In Fig. 4, the empirical convergence curves of the inner loop of Algorithm 1 for the cases of human and cat faces are depicted. The low-rank and sparse error matrices produced after 30, 50 and 117 iterations, respectively, are also shown.

### Feature-Based RSF (F-RSF)

In this section, we extend the RSF in order to be applied on images represented by multi-channel features, e.g, SIFT (Lowe 1999), HoGs (Dalal and Triggs 2005), IGOs (Tzimiropoulos et al. 2012) etc. The proposed extension is coined as Feature-based RSF (F-RSF). Given an input image $\mathbf{Q} \in \mathbb {R}^{h\times r}$ and a feature extraction function $\mathcal {K}: \mathbb {R}^{h\times r} \rightarrow \mathbb {R}^{h \cdot r \times G}$, the feature-based representation of the image is defined as $\mathbf{X}=[\mathbf{x}_1,\ldots , \mathbf{x}_G] \in \mathbb {R}^{h \cdot r \times G}$, where *G* is the number of the channels. Then, the problem of recovering the clean-frontal view in the feature space is formulated as follows:

20 $$\begin{aligned} \begin{aligned}&\underset{\{\{{\mathbf {L}}_j,\mathbf{c}_j,\mathbf{e}_j\}_{j=1}^G,{\varDelta }\mathbf{p}\},}{\mathop {\hbox {argmin }}\limits } \sum _{j=1}^G\Big (\left\| {\mathbf {L}}_j \right\| _* + \lambda \left\| \mathbf{e}_j \right\| _1\Big ) \\&\text {s.t.} \left\{ \begin{array}{ll} H^{(j,1)}({\varDelta }\mathbf{p}, \mathbf{c}_j, \mathbf{e}_j)=\mathbf{x}_j(\mathbf{p}) + \mathbf{J}_j(\mathbf{p}){\varDelta }\mathbf{p} - \mathbf{U}_j\mathbf{c}_j -\mathbf{e}_j={\mathbf {0}}\\ H^{(j,2)}({\mathbf {L}}_j,\mathbf{c}_j) = {\mathbf {L}}_j - \sum _{i=1}^k \mathcal {R}_{m \times n}(\mathbf{u}_{j,i}) c_{j,i}={\mathbf {0}}, \;\;\\ j = 1, 2,\ldots , G, \end{array}\right. \end{aligned}\nonumber \\ \end{aligned}$$

where ${\mathbf {L}}_j$ is the low-rank image, $\mathbf{c}_j$ is the linear combination coefficients, $\mathbf{e}_j$ is the sparse error, and $\mathbf{J}_j$ is the Jacobian for each channel $j=\{1,2,\ldots , G\}$. The shape parameters $\mathbf{p}$ and the corresponding increments ${\varDelta }\mathbf{p}$ are the same for all the channels. Furthermore, $\mathbf{U}_j$ are bases matrices computed using the *j* channel of expressionless clean frontal images. To minimize (20), the ADMM method is applied on the augmented Langragian:

21 $$\begin{aligned}&\mathcal {L}(\{{\mathbf {L}}_j,\mathbf{c}_j,\mathbf{e}_j,\mathcal {M}_j\}_{j=1}^G,{\varDelta }\mathbf{p}) = \sum _{j=1}^G\Big (\left\| {\mathbf {L}}_j \right\| _* + \lambda \left\| \mathbf{e}_j \right\| _1\Big ) \nonumber \\&\quad +\, \sum _{j=1}^G\Big (\frac{\mu }{2}\left\| H^{(j,1)}({\varDelta }\mathbf{p}, \mathbf{c}_j, \mathbf{e}_j) + \frac{\mathbf{a}_j}{\mu } \right\| _2^2 \nonumber \\ {}&\quad +\, \frac{\mu }{2}\left\| H^{(j,2)}({\mathbf {L}}_j,\mathbf{c}_j) + \frac{{\mathbf {B}}_j}{\mu } \right\| _F^2 -\frac{1}{2\mu }\left( \left\| \mathbf{a}_j \right\| _2^2 +\left\| {\mathbf {B}}_j \right\| _F^2 \right) \Big ), \end{aligned}$$

where $\mathcal {M}_j=\{\mathbf{a}_j, {\mathbf {B}}_j\}_{j=1}^G$ are the Lagrangian multipliers. Similarly to Algorithm 1, the proposed ADMM-based solver (outlined in Algorithm 2), minimizes (21) with respect to each variable in an alternating fashion and finally the Lagrange multipliers are updated at each iteration.

## Robust Face Frontalization in Videos

**Fig. 5:**
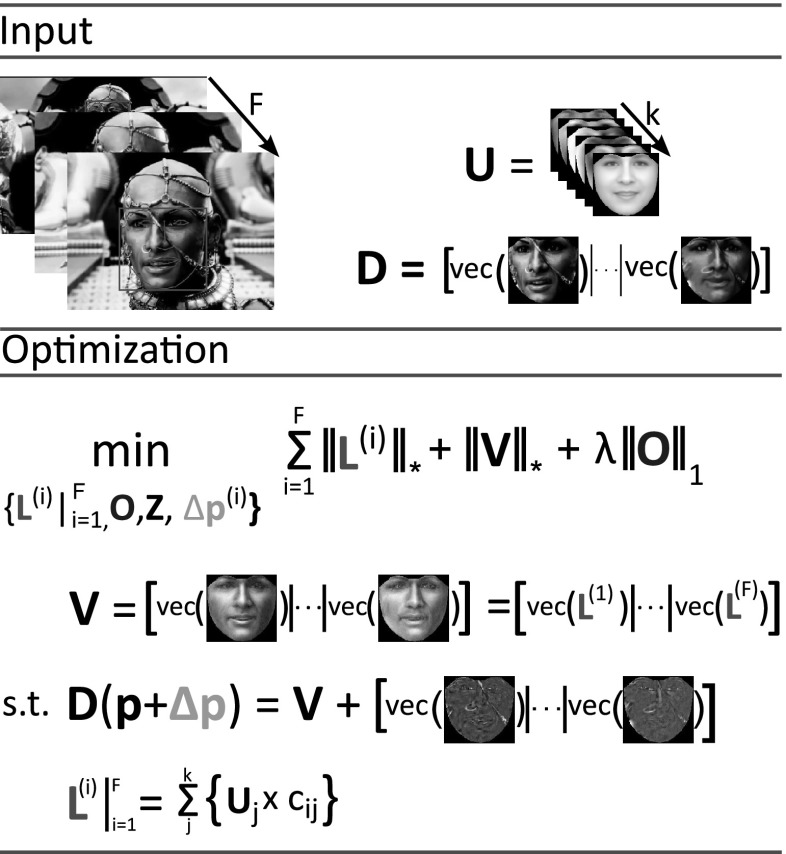
Robust Face Frontalization in Video: Given a video sequence consisting of *F* frames of the same subject, the results from a detector and a statistical model $\mathbf{U}$ a constrained low-rank minimization problem is solved. The frontal images, the increments of parameters, and sparse error matrices $\{{\mathbf {L}}, {\varDelta }\mathbf{p}, \mathbf{E}\}_{i=1}^F$ are computed subject the frontalized version of each frame is a low-rank image as well as the ensemble of all frontalized images is low-rank

Recognizing faces in videos is a task of paramount importance due to the wide range of commercial and surveillance applications. In recent years, the increasing popularity of commercial cameras, smart-phones, and video repositories such as Youtube has led to an increase of videos taken under uncontrolled (in-the-wild) conditions. The major problem in the recognition of a person in an in-the-wild video is that the appearance of the face dramatically changes under different poses, expressions, occlusions, and illumination conditions. In order to tackle these issues the method proposed in Sect. 3 can be applied independently in each frame of the video. Therefore, given a video sequence $\{\mathbf{X}^{(i)}\in \mathbb {R}^{h\times r}\}_{i=1}^F$ and the initial position of the landmarks in each frame the corresponding low-rank images $\{{\mathbf {L}}^{(i)}\in \mathbb {R}^{m\times n}\}_{i=1}^F$, sparse error matrices $\{\mathbf{E}^{(i)}\in \mathbb {R}^{m\times n}\}_{i=1}^F$ and corrected landmarks are produced. Then, the recognition can be performed by employing only the frontalized images $\{{\mathbf {L}}^{(i)}\}_{i=1}^F$ (Fig. 5).

However, by processing independently each frame rather than all frames together we do not take in consideration the temporal correlation among the frames. In case where all the frames are well-aligned the image ensemble ${\mathbf {D}}=[{{\mathrm{vec}}}(\mathbf{X}^{(1)}(\mathbf{p}^{(1)}))|\cdots | {{\mathrm{vec}}}(\mathbf{X}^{(F)}(\mathbf{p}^{(F)}))]\in \mathbb {R}^{m\cdot n \times F}$ lies in a low-rank subspace. By rectifying that fact, the problem of face frontalization in video can be formulated as follows:

22 $$\begin{aligned} \begin{aligned}&\underset{\{\{{\mathbf {L^{ (i)}}},{\varDelta }\mathbf{p}^{(i)}\}_{i=1}^F,\mathbf{O},{\mathbf {Z}}\}}{\mathop {\hbox {argmin }}\limits } \sum _{i=1}^F\left\| {\mathbf {L}}^{(i)}\right\| _* + \left\| \mathbf{V}\right\| _* + \lambda \left\| \mathbf{O} \right\| _1, \\&\text {s. t.} \left\{ \begin{array}{lll} G({\mathbf {D}},\{{\varDelta }\mathbf{p}^{(i)}\}_{i=1}^F\mathbf{V},\mathbf{O}) &{}=&{} {\mathbf {D}} - \mathbf{V} - \mathbf{O}\\ H^{(0)}(\mathbf{V},\{{\mathbf {L}}^{(i)}\}_{i=1}^F ) &{}=&{} \mathbf{V} - \sum _{i=1}^F {{\mathrm{vec}}}({\mathbf {L}}^{(i)})\mathbf{q}^{(i)^T}\\ H^{(i)}({\mathbf {L}}, \mathbf{C}) &{}=&{} {\mathbf {L}}^{(i)} \!-\! \sum _{j=1}^k \mathcal {R}_{m \times n}(\mathbf{u}_j) c_{ij}, \\ \end{array}\right. \end{aligned} \end{aligned}$$

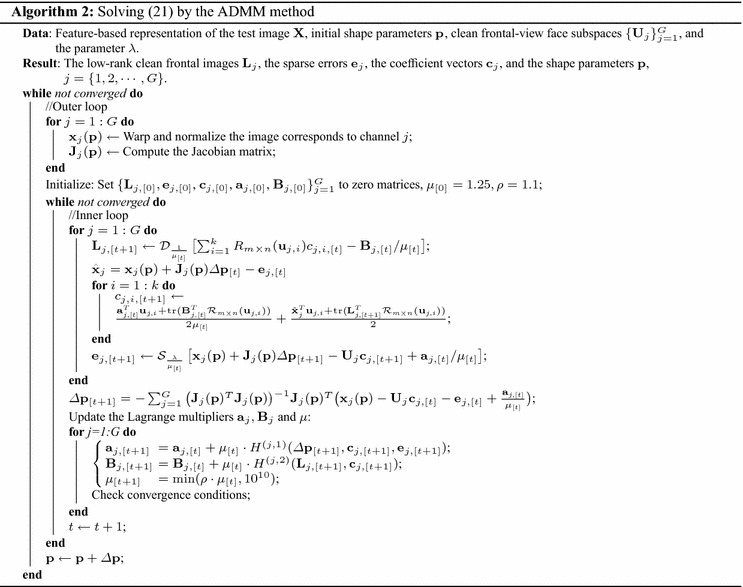

where $\mathbf{q}^{{(1)}^T},\mathbf{q}^{{(2)}^T},\ldots , \mathbf{q}^{{(F)}^T}$ are the standard bases of $\mathbb {R}^{F\times 1}$ and $\mathbf{O}\in \mathbb {R}^{m\cdot n \times F}$ is a sparse error matrix. To minimize (22), the ADMM is applied on the augmented Lagrangian:

23 $$\begin{aligned} \begin{aligned}&\mathcal {L}\left( \{{\mathbf {L^{ (i)}}},{\varDelta }\mathbf{p}^{(i)}, \mathbf{Y}^{(i)}\}_{i=1}^F,\mathbf{O},\mathbf{C}, \mathbf{M}\right) \!= \!\sum _{i=1}^F\left\| {\mathbf {L}}^{(i)}\right\| _* \!+\! \left\| \mathbf{V} \right\| _* \\&\quad +\,\lambda \left\| \mathbf{O} \right\| _1 + \frac{\mu }{2}\left( \bigg \Vert G({\mathbf {D}},\{{\varDelta }\mathbf{p}^{(i)}\}_{i=1}^F\mathbf{V},\mathbf{O}) + \frac{\mathbf{M}}{\mu }\bigg \Vert _F^2 \right. \\&\quad +\,\left\| H^{(0)} \big (\mathbf{V},\{{\mathbf {L}}^{(i)}\}_{i=1}^F\big ) + \frac{\mathbf{Y}^{(0)}}{\mu }\right\| _F^2 \\&\left. \quad +\,\sum _{i=i}^F\big \Vert H^{(i)}({\mathbf {L}}, \mathbf{C}) + \frac{\mathbf{Y}^{{(i)}}}{\mu } \big \Vert _F^2\right) \\&\quad -\,\frac{1}{2\mu } \left( \left\| \mathbf{M} \right\| _F^2 + \left\| \mathbf{Y}^{(0)} \right\| _F^2 + \sum _{i=1}^F \left\| \mathbf{Y}^{(i)} \right\| _F^2\right) , \end{aligned} \end{aligned}$$

yielding a similar to Algorithm 1 procedure. In (23), $\{\mathbf{M}, \mathbf{Y}^{(0)}\} \in \mathbb {R}^{m\cdot n \times F}, \{\mathbf{Y}^{(i)}\in \mathbb {R}^{m \times n}\}_{i=1}^F$ are the Lagrangian multipliers.

## Experimental Evaluation

The performance of the RSF is assessed in five different tasks: *(a) frontal view reconstruction*, *(b) landmark localization*, *(c) pose invariant face recognition*, *(d) face verification in unconstrained conditions*, and *(e) video inpainting* by conducting experiments in LFPW, (Belhumeur et al. 2011) HELEN (Le et al. 2012), AFW (Zhu and Ramanan 2012), FERET (Phillips et al. 2000), Multi-PIE (Gross et al. 2010), LFW (Huang et al. 2007), FS (Zhang et al. 2011b; Wang and Tang 2009), and CAT (Zhang et al. 2008) databases. Furthermore, the YTF (Wolf et al. 2011) database is employed in order to evaluate the performance of RSF-V for the video face verification task.

### Data Description

Let us first provide a brief description of the databases used in the evaluation studies.

*LFPW:* The Labeled Faces Parts in-the-wild (LFPW) (Belhumeur et al. 2011) database contains face images downloaded from the internet (i.e., gooogle.com, flickr.com etc). The images depict multiple variations of faces in terms of pose, expression, illumination, and occlusions. Since only the URLs of images were provided, 811 out of the 1132 training images and 224 out of the 300 test images were downloaded.

*HELEN:* The HELEN (Le et al. 2012) database consists of 2330 face images (2000 train, 330 test) downloaded from Flickr web service. A broad range of face appearance variations, including pose, lighting, expression, occlusion, and individual differences are depicted in these images.

*AFW:* The Annotated Faces in-the-wild (AFW) (Zhu and Ramanan 2012) database consists of 250 images with 468 faces. That is more than one face is annotated in each image. The images depict similar facial variations as those in the LFPW and HELEN databases.

*FERET:* The Facial Recognition Technology (FERET)(Phillips et al. 2000) database consists of 14051 images of 200 different subjects. All images capture the same ‘Neutral’ expression for 9 different head poses under different illuminations. Each subject also has an additional image of a random facial expression.

*Multi-PIE:* The CMU Multi Pose Illumination and Expression (Multi-PIE) (Gross et al. 2010) database consists of approximately 750,000 images from 337 subjects, captured under 6 different expressions, 15 poses, and 19 illuminations.

*LFW:* The Labeled Faces in the Wild (LFW) (Huang et al. 2007) database contains 13,233 images of 5749 people downloaded from the Web and is designed as a benchmark for the problem of unconstrained automatic face verification. All images are characterized by large variations in pose, expression and occlusion.

*YTF:* The Youtube Face database (YTF) is considered as the basic benchmark for video-based unconstrained face verification. It consists of 3425 videos of 1595 subjects acquired from Youtube. In average, the are 2.15 videos available for each subject, while each video contains 181.3 frames. Similar variations with those in the LFW are appeared in frames.

*FS:* The CUHK Fase Sketch (CUFS)(Wang and Tang 2009) and CUHK Face Sketch FERET Database (CUFSF) (Zhang et al. 2011b) contains 606 and 1194 face sketches, respectively. Each sketch is drawn by an artist based on a face image captured normal lighting conditions, in frontal pose while being expressionless. A set of 375 images (305 images taken from the above databases and another 53 images download from the web) were employed in the experiments. All images were annotated in terms of 68 landmark points.

*CAT:* The CAT (Zhang et al. 2008) database consists of 10, 000 cat images obtained from flickr.com. Annotations regarding 9 points for each cat head are provided. A subset of 350 images was used in the conducted experiments. The selected images were re-annotated by employing a dense mark-up scheme consisting of 48 points (Sagonas et al. 2015).

### Experimental Setup

In all the experiments, the orthonormal clean frontal subspace $\mathbf{U}$ was constructed by employing only frontal view face images without occlusions. The images were warped in a reference frame by using the $\mathcal {W}$ (cf. Sect. 2). Subsequently, PCA was applied on the warped shape-free textures. Then, the first *k* eigen-images with the highest variance were used to form the $\mathbf{U}$. In Table 1, information regarding the construction of $\mathbf{U}$, as used in our experimental evaluation, are provided.

### Reconstruction of Frontal View

The ability of the RSF to reconstruct the frontal view from non-frontal images of unseen faces is investigated in this section. Given the test image and initial landmarks a warped version of the image is produced by employing the $\mathcal {W}$. Next, (3) is solved iteratively. In each iteration $t+1$, a low-rank (frontalized) image (${\mathbf {L}}_{[t+1]}$), an error sparse error matrix ($\mathbf{E}_{[t+1]}$), coefficients ($\mathbf{c}_{[t+1]}$) and increments ${\varDelta }\mathbf{p}_{[t+1]}$ of parameters $\mathbf{p}$ are obtained. The new position of the landmarks is then computed by employing the updated parameters $\mathbf{p}$ ($\mathbf{p}\leftarrow \mathbf{p} + {\varDelta }\mathbf{p}_{[t+1]}$). The test image is then warped using the new landmarks and  (3) is solved again (INNER loop of Algorithm 1). Finally, after the convergence of Algorithm 1, the final frontalized test image, location of the landmarks, and error sparse error matrix are produced. All the frontalization presented in this Section were created by using the $\mathbf{U}_{\text {W}}$, $\mathbf{U}_{\text {C}}$, and $\mathbf{U}_{\text {S}}$.

**Table 1:** Definition of the clean frontal subspaces $\mathbf{U}_\text {W}$, $\mathbf{U}_\text {L}$, $\mathbf{U}_\text {H}$, $\mathbf{U}_\text {C}$ and $\mathbf{U}_\text {S}$

	# Images	Source	Reference frame	*k*
$\mathbf{U}_{\text {W}}$	587	LFPW & HELEN	$184 \times 193$	450
$\mathbf{U}_{\text {L}}$	209	LFPW	$184 \times 193$	200
$\mathbf{U}_{\text {H}}$	284	HELEN	$184 \times 193$	250
$\mathbf{U}_{\text {S}}$	305	FS	$184 \times 193$	300
$\mathbf{U}_{\text {C}}$	261	CAT	$243 \times 233$	260

Frontal images without occlusions are selected from the training set of the used databases

Unless otherwise stated, throughout the experiments, the parameters of the Algorithm 1 were fixed as follows: $\lambda = 0.3$, $\rho = 1.1$, $\epsilon _1=10^{-5}$, $\epsilon _2=10^{-7}$, and $\epsilon _3=10^{-3}$.

**Fig. 6:**
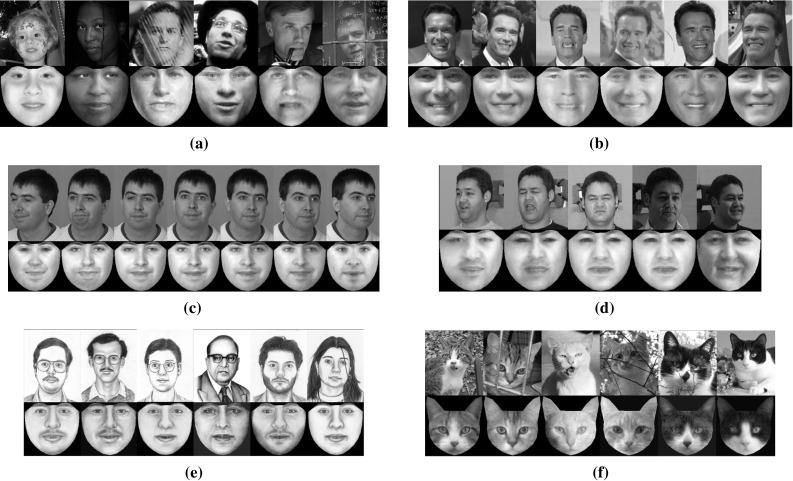
Reconstructed frontal views of unseen subjects under controlled (Multi-PIE, FERET, SK) and unconstrained conditions (LFPW, HELEN, AFW, LFW, CAT). The frontalization **a**–**f** were obtained by employing the $\mathbf{U}_W$, $\mathbf{U}_C$, and $\mathbf{U}_S$, respectively. **a** LFPW—HELEN—AFW, **b** LFW, **c** FERET, **d** Multi-PIE, **e** SK, **f** CAT

In Fig. 6a, b the frontalized views of unseen faces from the LFPW, Helen, AFW and LFW databases are illustrated. Figure 6c, d depict the frontal reconstructed views from the non-frontal images of subject with id ‘00268’ from FERET and images from Multi-PIE with (a) ‘Surprise’ at $-30^\circ$, (b) ‘Scream’ at $-15^\circ$, (c) ‘Squint’ at $0^\circ$, (d) ‘Neutral’ at $+15^\circ$, and (e) ‘Smile’ at $+30^\circ$. The efficacy of the RSF is also assessed by creating the frontal view of face sketches and cat faces. The obtained reconstructions for these objects are depicted in Fig. 6e, f. By visually inspecting the results, it is clear that the RSF is robust to many variations such as pose, expression, and sparse occlusions. This attributed to the fact that the matrix $\ell _1$-norm was adopted for sparse non-Gaussian noise characterization.

In order to assess the effectiveness of the RSF in handling different illumination conditions, we conducted the following experiment. We selected ‘Neutral’ images of three subjects from the Multi-PIE database under poses $-15^\circ$ to $15^\circ$. For each pose and subject, 11 images captured under 11 different illumination conditions were used. Then, the images of each subject (30 in total) were frontalized by employing the RSF with the basis matrix $\mathbf{U}_{\text {W}}$. The obtained frontalized views of all subjects are depicted in Fig. 8. As it can been observed, the RSF reconstructs successfully the frontal view of the unseen subject and in most of cases removes the illumination effects.

**Fig. 7:**
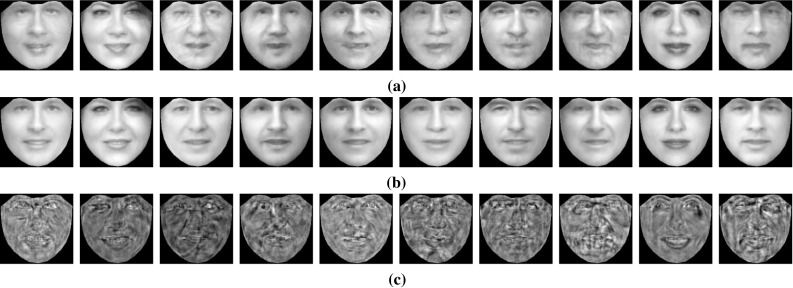
Qualitative evaluation of the reconstructed frontal views. The quality of the results obtained by the RSF can be assessed from the averages of images of 10 subjects from the CACD (Chen et al. 2014) database before and after frontalization. **a** Average warped input images. **b** Average recovered frontal view images. **c** Average sparse error matrices

**Fig. 8:**
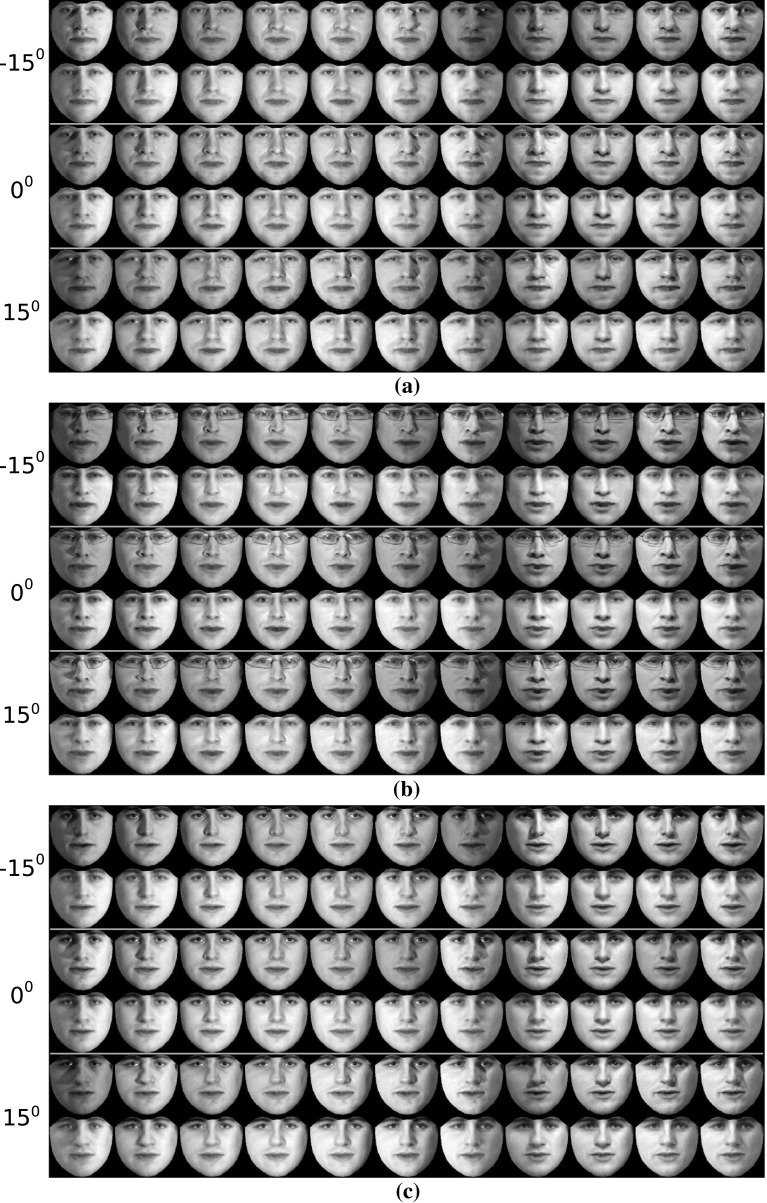
Reconstructed frontal view of unseen subjects under 11 different illumination conditions and poses $-15^\circ : 15^\circ$. The first row in each pose corresponds to the warped input image, while the second one to the frontalized view. **a** Subject 1. **b** Subject 2. **c** Subject 3

As an additional example, 100 images (10 images for each subject) of 10 subjects from CACD database (Chen et al. 2014) were frontalized by employing the Algorithm 1. In Fig. 7 the averages of input, frontalized, and sparse error matrices are depicted. As it can been observed, the averages of faces after frontalization are much sharper, and detailed than the average input images, indicating the frontalization quality achieved by the RSF.

To quantitatively assess the quality of the frontalized images the following experiment was conducted. ‘Neutral’ images of 20 different subjects from Multi-PIE under poses $-30^\circ$ to $30^\circ$ (5 for each subject, 100 in total) were selected. The images of each subject were frontalized by employing the RSF. The Root Mean Square Error (RMSE) between each frontalized image and the real frontal image of the subject is used as the evaluation metric. The average RMSE of the RSF is 0.0817. The performance of the RSF with respect to RMSE is compared with that obtained by the frontalization method of the DeepFace (Taigman et al. 2014) which achieved an average RMSE of 0.1025. It is worth noting that, even though DeepFace employs a 3D model to handle out-of-planar rotations, the RSF performs better without using any kind of 3D information.

**Fig. 9:**
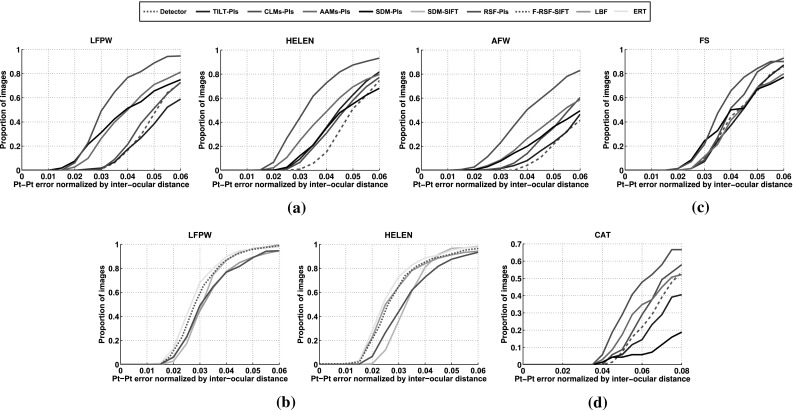
Cumulative error distribution *curves* on LFPW, HELEN, AFW, FS, and CAT databases. **a**, **c**, **d** TILT-PIs, CLMS-PIs, AAM-PIs, SDM-PIs, RSF-PIs, **b** RSF-PIs, SDM-SIFT, LBF, ERT and F-RSF-SIFT

### Landmark Localization

The performance of the RSF for the generic alignment problem is assessed by conducting experiments on (a) in-the-wild faces, (b) sketch faces and (c) cat faces. To this end, the performance of the RSF is compared to that obtained by the TILT (Zhang et al. 2012), AAMs (Matthews and Baker 2004), CLMs (Saragih et al. 2011), and SDM (Xiong and De la Torre 2013). In order to fairly compare the competing methods, the same training data (the same images which were used to build the $\mathbf{U}_\text {W}$), initialization, and feature representation were employed. For all experiments the simple representation of pixel intensities (PIs) was used. The average point-to-point Euclidean distance of *N* landmark points normalized by the Euclidean distance of the outer corner of eyes is used as the evaluation measure. More specifically, by denoting the ground truth and fitted shapes of an image *i* as $\mathbf{s}_{gt}$ and $\mathbf{s}_f$ respectively and the Euclidean distance between the outer corners of the eyes as $d_{outer}$, the fitting error is given by:

24 $$\begin{aligned} e_f = \frac{\sqrt{\sum _{j=1}^N (x^{(j)}_{gt} - x^{(j)}_{f})^2 + (y^{(j)}_{gt} - y^{(j)}_{f})^2}}{d_{outer}N}. \end{aligned}$$

In case of human faces the outer corners of eyes are the $[x^{(37)},y^{(37)}]$, $[x^{(46)},y^{(46)}]$, and the normalization distance is defined as $d_{outer} = \sqrt{(x_{gt}^{(37)} - x_{gt}^{(46)})^2 + (y_{gt}^{(37)} - y_{gt}^{(46)})^2}$, while in case of cats the outer corners of eyes are the $[x^{(33)},y^{(33)}]$, $[x^{(42)},y^{(42)}]$ and $d_{outer} = \sqrt{(x_{gt}^{(33)} - y_{gt}^{(42)})^2 + (x_{gt}^{(33)} - y_{gt}^{(42)})^2}$. In addition, the cumulative error distribution curve (CED) for each method was computed by using the fraction of test images for which the average error was smaller than a threshold. Finally, the implementations provided by the platform MENPO (Alabort-i Medina et al. 2014) were used for all compared methods.

**Table 2:** Fitting performance on LFPW, HELEN, AFW, FS, and CAT databases using TILT-PIs, CLMs-PIs, AAMs-PIs, SDM-PIs, and RSF-PIs: Proportion of images with normalized error $<\{0.02, 0.03,0.04,0.05,0.06\}$

Database	LFPW			HELEN			AFW			FS			CAT		
Method	0.02	<0.03	<0.05	<0.02	$<0.03$	<0.05	<0.02	<0.03	<0.05	<0.02	<0.03	<0.05	<0.03	<0.04	<0.06
TILT-PIs	0.00	1.79	38.39	0.00	9.39	62.73	0.00	1.48	23.44	0.00	7.14	68.57	0.00	1.45	14.49
CLM-PIs	0.00	0.89	51.34	0.30	6.67	58.18	0.00	1.78	35.31	0.00	8.57	81.43	0.00	0.00	28.99
AAM-PIs	2.68	26.34	70.98	2.12	24.85	69.39	0.59	8.61	43.32	0.00	11.43	68.57	0.00	2.90	34.78
SDM-PIs	**7**.**59**	31.25	65.63	0.00	12.12	55.15	0.30	7.72	36.20	**1**.**43**	**24**.**29**	67.14	0.00	1.45	5.80
RSF-PIs	6.25	**49**.**11**	**88**.**84**	**6**.**67**	**43**.**94**	**87**.**58**	**2**.**67**	**23**.**15**	**68**.**25**	**1**.**43**	21.43	**84**.**29**	0.00	**5**.**80**	**47**.**83**

#### Aligning in-the-Wild Face Images

The in-the-wild face databases LFPW, HELEN and AFW were employed in order to assess the performance of the RSF in the problem of generic face alignment. The results produced by the detector in (Zhu and Ramanan 2012) were used to initialize all the methods. The annotations provided in (Sagonas et al. 2013b, a, 2016) have been employed for evaluation purposes. The error for each method was computed based on $N=49$ interior landmark points (excluding the points correspond to face boundary). Finally, the bases matrices $\mathbf{U}_{\text {L}}, \mathbf{U}_{\text {H}}$ and $\mathbf{U}_{\text {W}}$ were used by the RSF.

**Table 3:** Average time, in CPU seconds, required from the competing methods to fit one image

TILT-PIs	CLMS-PIs	AAMS-PIs	SDM-PIs	RSF-PIs
45	0.4	0.6	0.05	30

**Fig. 10:**
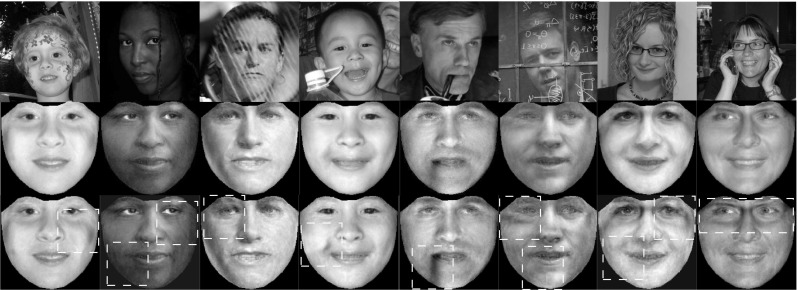
Reconstructed frontal view of unseen subjects under unconstrained conditions: $1^{st}$-row) input images, $2^{nd}$-row) $\ell _1$-RSF-PIs, and $3^{rd}$-row) $\ell _2$-RSF-PIs

The CEDs produced by all methods for the LFPW (test set), the HELEN (test set), and the AFW databases are depicted in Fig.  9a. Clearly, the RSF outperforms the TILT-PIs, the AAMs-PIs, the CLMs-PIs, and the SDM-PIs. More specifically, for normalized error of 0.05 the RSF yield an 20.1, 21.5 and 24.6 % improvement compared to that obtained by the AAMs-PIs in the LFPW, HELEN and AFW databases, respectively. TILT performs worst overall which can be explained by the fact that it minimizes the unconstrained rank of the image ensemble. The discriminative methods SDM and CLMs yield poor performance because they were trained with only 500 frontal images. In general the discriminative methods require large amount of annotated data in order to yield powerful classifiers and functional mappings. In contrast, AAMs which are generative models, achieved better results than the CLMs and SDM. In Table 2 the proportion of images with normalized error lower than 0.02, 0.03, and 0.05 for the competing methods are reported. A few fitting examples from the test databases are depicted in Fig.  12. Furthermore, we computed the average time, in CPU seconds, that each method requires to fit one image. By inspecting Table 3 we observe that the CLM, AAMs, and SDM are faster than the RSF. This is attributed to the high computational complexity of Singular Value Decomposition in each step of the RSF (cf. Algorithm 1, Step 1). The computational complexity of the RSF can be reduced by using fast variants of the Singular Value Thresholding operator e.g., (Cai and Osher 2010; Oh et al. 2015), in order to solve the nuclear norm regularized least squared problem (10). However, such modification is out of the scope of our paper.

We also compared RSF to the state-of-the-art methods SDM (Xiong and De la Torre 2013), LBF (Ren et al. 2014), and ERT (Kazemi and Sullivan 2014). The authors provided pre-trained model and code was used for the SDM, while the LBF and ERT were trained and tested by using the available implementations.^4^ In particular, the LBF and ERT were trained using the AFW and train sets of LFPW and HELEN. The parameters were set as explained in corresponding papers. The CEDs from this experiment are shown in Fig. 9b. The RSF achieves comparable performance with that obtained by the competing methods, but it uses only a small set of frontal images for training. This is in contrast to all other methods that were trained on thousand images captured under several variations including different poses, illuminations and expression (i.e., train sets of the used databases). Furthermore, the SDM method takes full advantage of SIFT—a powerful hand-crafted feature—while the RSF employs only simple PIs. Figure 12a illustrates fitting examples produced by RSF.

**Fig. 11:**
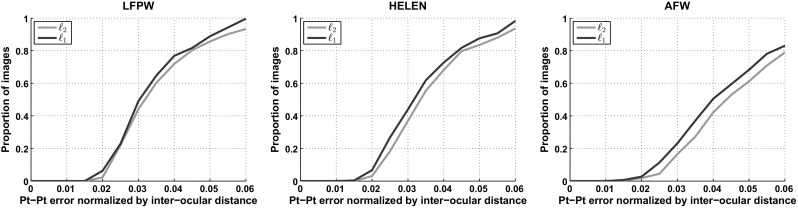
Cumulative error distribution curves on LFPW, HELEN, AFW databases. Compared methods: $\ell _1$-RSF-PIs and $\ell _2$-RSF-PIs

The performance of the F-RSF on generic face alignment is also assessed by conducting experiments on the LFPW and HELEN databases. To this end, the same initializations and procedure described before was followed. The dense-SIFT features with $G=36$ channels were used by the F-RSF. In order to build the basis matrices $\mathbf{U}_j, j=\{1, 2, \ldots , G\}$ we computed the dense SIFT features of the clean frontal images and then the images correspond to each channel *j* were used to compute the $\mathbf{U}_j$. The performance of the F-RSF is compared against that obtained by the RSF-PIs and state-of-the-art methods SDM, LBR, and ERT. The CEDs produced by the competing methods are presented in Fig. 9b. As it can been seen the F-RSF outperforms the RSF-PIs, SDM, and LBF while performs very closely to the state-of-the-art method ERT.

Even though, the intrinsic motivation of the RSF is to deal with gross, but sparse, non-Gaussian noise that often appears in face image acquired under real world conditions (e.g., device artifacts such as pixel corruptions, missing and incomplete data such as partial image texture occlusions, or localization errors). The RSF can implicitly handle data contaminated by Gaussian noise by vanishing the error term. That is by setting the weighting parameter in optimization problem (2) $\lambda \rightarrow \infty$, i.e. $\mathbf{E}={\mathbf {0}}$. In this case, the $\ell _2$ norm $\frac{\mu }{2} || H^{(1)}({\varDelta }\mathbf{p},\mathbf{c})||_2^2$ appearing in the augmented Lagrangian function (5) is deemed as the appropriate regularized for handling Gaussian noise.

The effectiveness of the RSF-PIs under Gaussian noise is assessed in face frontalization and landmark localization. In both experiments the parameter $\lambda$ was set equal to 10000. In Fig. 10 the frontalized faces obtained by the $\ell _1$-RSF-PIs and $\ell _2$-RSF-PIs using the $\mathbf{U}_\text {W}$ are depicted in rows 2 and 3, respectively. As it can been seen the faces produced by the $\ell _2$-RSF-PIs are more noisy than those produced by $\ell _1$-RSF-PIs. More specifically, in cases where the face is partially occluded (please see inside the red dotted boxes) the $\ell _2$-RSF-PIs fails to remove the occlusion and introduces noise to the non-concluded area of the face. In addition, we assess the effectiveness of the $\ell _2$-RSF-PIs in the problem of landmark localization by conducting experiments in the LFPW, HELEN, and AFW databases. The same parameters, procedure, and metric errors as before were used in this experiment. In Fig. 11 the CEDs correspond to the results obtained by the $\ell _1$-RSF-PIs and $\ell _2$-RSF-PIs are depicted. The results demonstrate that the $\ell _1$-RSF-PIs outperforms the $\ell _2$-RSF-PIs in all databases. The obtained results in both face frontalization and landmark localization indicate the superiority of usage of the $\ell _1$ norm.

**Fig. 12:**
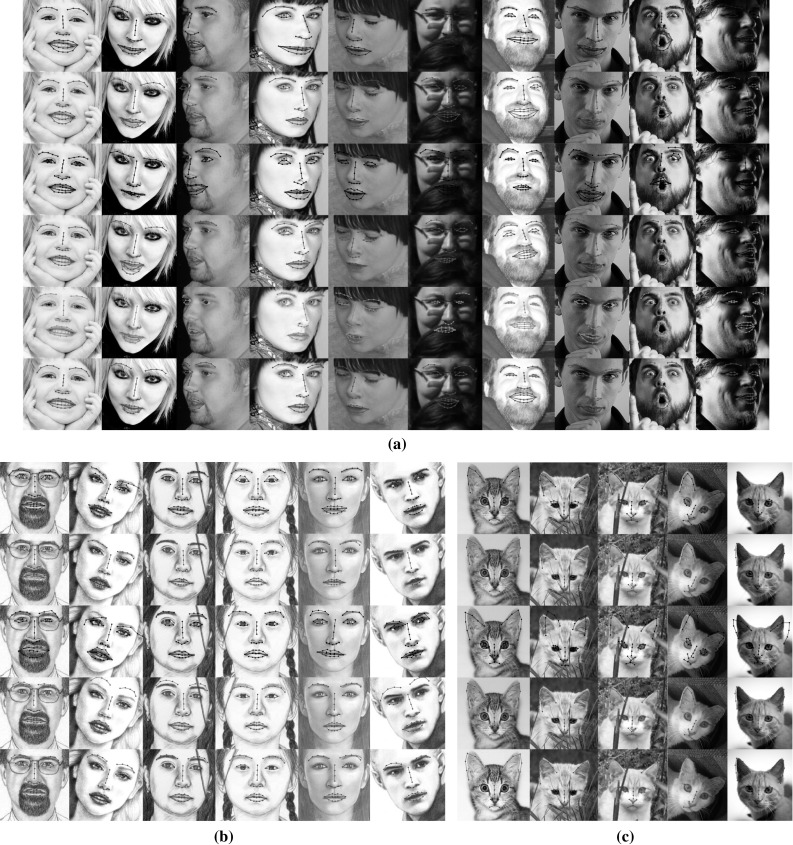
Sample fitting results produced by the compared methods: TILT-PIs (*blue*), CLMs-PIs (*green*), SDM-PIs (*black*), AAMs-PIs (*magenta*), SDM-SIFT (Xiong and De la Torre 2013) (*cyan*), RSF (*red*). **a** human faces, **b** face sketches, **c** cat faces

#### Aligning Cat and Sketch Face Images

RSF is a general technique and we demonstrate that by its ability to align face sketches and cat faces. To this end, we use the FS and CAT databases. The matrices $\mathbf{U}_\text {C}$, $\mathbf{U}_\text {S}$ were employed and the fitting error in case of CAT was calculated based on $N = 37$ interior landmark points (excluding the points of boundary). The results obtained by the compared methods are summarized in Fig. 9c, d and Table 2. The quality of fitting results produced by the methods can be seen in Fig. 12. The RSF outperforms all other methods, and demonstrates the ability to handle any face-like objects.

### Pose-Invariant Face Recognition 

The performance of the RSF on pose invariant face recognition with one gallery image per person is assessed by conducting experiments on the Multi-PIE and FERET databases. The experiment proceeds as follows. First, the frontal views of all images used in this experiment were reconstructed following the methodology described in Sect. 5.3 by employing the $\mathbf{U}_\text {W}$. In order to remove the surrounding black pixels, the reconstructed frontal views were cropped. Subsequently, the Image Gradient Orientations (IGOs) features (Tzimiropoulos et al. 2012) were used for image representation. Let us denote an image in vectorial form as $\mathbf{v}$ with size $d \times 1$, thus *d* is the number of pixels. Moreover, $\mathbf{g}_x,\mathbf{g}_y$ denote the image gradients and $\phi = \arctan (\mathbf{g}_x/\mathbf{g}_y)$ the corresponding gradients orientation vector. The normalized gradients extraction function $\mathcal {F}: \mathbb {R}^{d\times 1} \rightarrow \mathbb {R}^{2d \times 1}$ is defined as

25 $$\begin{aligned} \mathcal {F} = \frac{1}{d}[\cos ({{\mathbf {\phi }}})^T , \sin ({{\mathbf {\phi }}})^T ]^T, \end{aligned}$$

where $\cos ({\phi }) = [\cos ({\phi }(1)),\ldots , \cos ({\phi }(d))]$ and $\sin ({\phi }) = [\sin ({\phi }(1)),\ldots , \sin ({\phi }(d))]$. The dimensionality of IGOs was reduced by applying PCA. Finally, the classification was performed by employing the Collaborative Representation based Classifier (CRC) in (Zhang et al. 2011a).

The performance of the RSF is compared to 2D based methods: LGBP (Zhang et al. 2005) and PIMRF (Ho and Chellappa 2013), 3D based methods: 3DPN (Asthana et al. 2011), EGFC (Li et al. 2012b), and PAF (Yi et al. 2013), as well as the Deep learning based methods: SPAE (Kan et al. 2014) and DIPFS (Zhu et al. 2013). It should be noticed that all methods were evaluated under the fully automatic scenario, where both the bounding box of the face region and the facial landmarks were located automatically.

#### Results on FERET

**Fig. 13:**
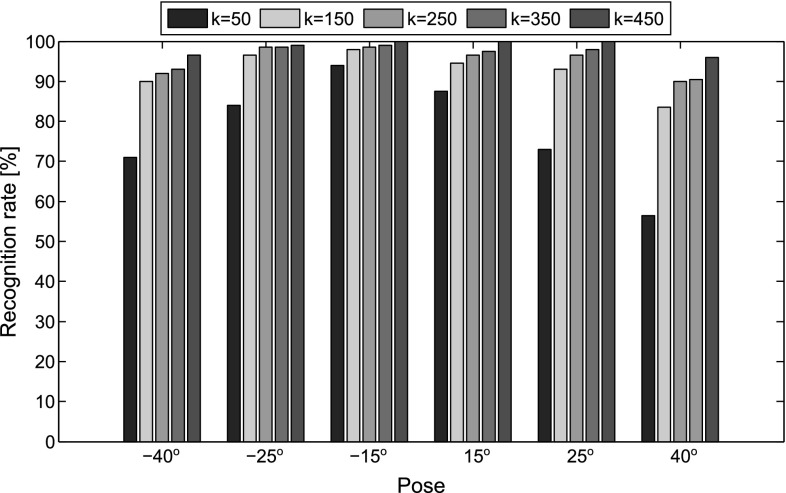
Recognition accuracy of RSF in FERET database for different number of eigen-images *k*

One frontal image, denoted as ‘ba’, from each of the 200 subjects was used to form the gallery set, while the images captured at 6 different poses i.e., $-40^\circ$ to $40^\circ$ were selected as the probe images. Before comparing RSF with existing methods, the impact of number of eigen-images, i.e, *k* in recognition performance was investigated. To this end, the clean frontal subspace $\mathbf{U}_W$ with $k \in \{50,150,250,350,450\}$ was used in order to frontalize the images. Figure 13 shows the recognition accuracy obtained for each *k*. It is clear that the more eigen-images are used the better the performance. In particular, a steep improvement is observed in large poses such as $-40^\circ$ and $40^\circ$. The self-occlusions appearing in large poses result in high variability of the textures in these cases, which explains why using more eigen-images leads to an improve to performance.

**Table 4:** Recognition rates ($\%$) achieved by the compared methods on the FERET database

Method	bh	bg	bf	be	bd	bc	Avg
	$-40^\circ$	$-25^\circ$	$-15^\circ$	$+15^\circ$	$+25^\circ$	$+40^\circ$	
LGBP	62.0 %	91.0 %	98.0 %	96.0 %	84.0 %	51.0 %	80.5 %
3DPN	90.5 %	98.0 %	98.5 %	97.5 %	97.0 %	91.9 %	95.6 %
PIMRF	91.0 %	97.3 %	98.0 %	98.5 %	96.5 %	91.5 %	95.5 %
PAF	98.0 %	98.5 %	99.25 %	99.25 %	98.5 %	98.0 %	98.56 %
RSF	96.5 %	99.0 %	100.0 %	100.0 %	100 %	96 %	98.58 %

**Table 5:** Recognition rates ($\%$) achieved by the compared methods on the Multi-PIE database

Method	$130\_06$	$140\_06$	$051\_07$	$050\_08$	$041\_08$	Avg
	$-30^\circ$	$-15^\circ$	$0^\circ$	$15^\circ$	$30^\circ$	
PIMRF	89.7 %	91.7 %	92.5 %	91.0 %	89.0 %	90.78 %
3DPN	91.0 %	95.7 %	96.9 %	95.7 %	89.5 %	93.76 %
SPAE	92.6 %	96.3 %	–	95.7 %	94.3 %	94.72 %
EGFC	95.0 %	99.3 %	–	99.0 %	92.9 %	96.55 %
DIPFS	98.5 %	100 %	–	99.3 %	98.5 %	99.07 %
RSF	94.3 %	98.7 %	99.4 %	97.3 %	95.6 %	97.06 %

In Table 4 the recognition rates achieved by the competing methods in the different poses are reported. Clearly, the RSF (recognition accuracy 98.58 %) outperforms both the 2D and 3D state-of-the-art methods. It is worth mentioning that the PIMRF employs 200 images from the FERET database (different from the test set) in order to train the frontal synthesizer. Consequently, the different lighting conditions of the database are taken into account. This is not the case for the RSF where only frontal images from a generic in-the-wild database (i.e., the LFPW and HELEN) have been used. Even though the RSF does not use any kind of 3D information, it performs comparably to the PAF where an elaborated 3D model (trained from 4624 facial scans) has been used.

#### Results on Multi-PIE

The images of 137 subjects (Subject ID 201: 346) with ‘Neutral’ expression and poses $-30^\circ$ to $+30^\circ$ captured under 4 different sessions were selected. The gallery was created by the frontal images of the earliest session for each subject, while the rest of images including frontal and non-frontal views were used as probes. It should be mentioned that images of first 200 subjects which include all poses (4207 in total) were not used for training purposes. Those images were used in the 3DPN to train view-based models, in the SPAE, DIPFS to train the deep neural networks, and in the EGFC to train the pose estimator and matching model. The recognition accuracy achieved by the compared methods is reported in Table 5. The RSF outperforms four out of five methods that is compared to. The RSF also performs comparable to the DIPFS though only using 500 frontal images outside the Multi-PIE. It should be noticed that in DIPFS the positions of eyes which were used to align both the train and test images were located manually. In contrary RSF is a fully automatic method and all the landmarks were automatically detected. Furthermore, the $\mathbf{U}_{\text {LFW}}$ used by the RSF was built by images outside the Multi-PIE, while only images from Multi-PIE used by DIPFS to build the deep-learning feature extractor.

### Face Verification in Unconstrained Conditions

#### Image Face Verification on LFW Database

**Fig. 14:**
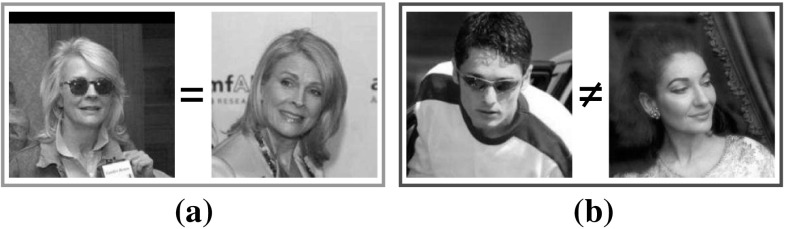
Sample image pairs from LFW database with **a** same and **b** different persons, respectively

The performance of the RSF in the face verification under in-the-wild conditions is assessed by conducting experiment in the LFW database, using the *image-restricted, no outside data results* setting. The standard evaluation protocol, which splits the View 2 dataset into 10 folds, with each fold consisting of 300 intra-class pairs and 300 inter-class pairs, was employed. In Fig. 14 sample images pairs of the same and different persons are depicted. As it can been seen in the case of same pair there is a big change in appearance of the subject (different pose and illumination conditions, sunglasses).

**Table 6:** LFW: Mean classification error and standard deviation

LFW3D-IGOs-SVM	$0.7928 \pm 0.0175$
MRF-MLBP	$0.7908 \pm 0.0014$
APEM-SIFT	$0.8188 \pm 0.0094$
Eigen-PEP	$0.8627 \pm 0.0106$
Eigen-PEP (flip)	$0.8897 \pm 0.0132$
MRF-MLBP-CSDKDA	$0.9068 \pm 0.0132$
POP-PEP-SIFT	$0.9110 \pm 0.0147$
Spartans	$0.8755 \pm 0.0021$
APEM-Fusion	$0.8408 \pm 0.0120$
Fisher vector faces (flip)	$0.8747 \pm 0.0149$
MRF-Fusion-CSDKDA	$0.9589 \pm 0.0194$
RSF	$0.8881 \pm 0.0078$

In this experiment the basis $\mathbf{U}_{\text {W}}$ and the detector in (Zhu and Ramanan 2012) were not used since they are based on images outside the database. To create the initializations and a new $\mathbf{U}_\text {LFW}$, the method for automatic construction of deformable models presented in (Antonakos and Zafeiriou 2014) was employed. The goal of this method is to build a deformable model using only a set of images with the corresponding face bounding boxes. To define the face bounding boxes without using a pre-trained detector, the deep funneled images of the LFW (Huang et al. 2012) were employed. Therefore, since these images are aligned, the exact face bounding box is known. Subsequently, a deformable model was built automatically from the training images of each fold. The created model was fitted to all images and those (from training images) with fitted shapes similar to the mean shape were selected to build the basis $\mathbf{U}_\text {LFW}$. In each fold the images were frontalized using the $\mathbf{U}_\text {LFW}$ and they were cropped subsequently. The gradient orientations $\phi _1$, $\phi _2$ of each image pair were extracted and the cosine of difference between them ${\varDelta }\phi =\phi _1-\phi _2$ was normalized to the range $[0-2\pi ]$, and used as the feature of the pair. In order to reduce the dimensionality of the features a PCA, computed from the train folds each time, was applied. These features are classified by a support vector machine (SVM) with an RBF kernel. The performance of the RSF is compared against that obtained by methods which use single descriptor and without augmenting the training set with flipped images. To this end, the MRF-MLBP (Arashloo and Kittler 2013), APEM-SIFT (Li et al. 2014), Eigen-PEP (Li et al. 2014), MRF-MLBP-CSKDA (Arashloo and Kittler 2014), POP-PEP-SIFT (Li and Hua 2015) and Spartans (Juefei-Xu et al. 2015) were selected for comparisons. The mean classification accuracy and the corresponding standard deviation computed based on 10 folds are reported in Table 6. In order to make the Table self-contained the results achieved using multiple descriptors and flipped training images are also reported. By inspecting Table 6, it can be seen that the RSF outperforms the APEM-SIFT, MRF-MLBP, Eigen-PEP, and the Spartans and performs comparably to the recently published MRF-MLBP-CSDKA and POP-PEP. It is worth mentioning that, the MRF-MLBP-CSDKDA employs an MRF, which has computationally heavy optimization, for dense image matching followed by multi-scale features extraction. In addition, the POP-PEP model which has a deep architecture (consists of 3 layers), requires 41 hours for training and uses SIFT-a powerful handcrafted feature. In contrary, the RSF is more computationally efficient since IGOs, computed in one scale are employed.

Recently, a new frontalization version of the LFW named LFW3D has been proposed in (Hassner et al. 2015). In order to compare the quality of frontalizations between the RSF and LFW3D, the same classification framework as before was applied on LFW3D. The achieved accuracy is 79.28 % while the accuracy achieved by the RSF is 88.81 %. This is a quite interesting result since the proposed RSF method does not use any kind of 3D information. This is due to the fact that in RSF sparse noise such as occlusions and illuminations is removed from the frontalized images.

#### Video Face Verification on YouTube Faces Database

The YTF (Wolf et al. 2011) was employed in order to assess the performance of the RSF-V in the problem of video-based face verification. The standard *restricted* evaluation protocol of 10 folds, with each fold consisting of 250 intra-class and 250 inter-class pairs, was adopted. The experiment proceeds as follows. First, the RSF-V was employed in order frontalize the frames of each video. Then, the mean appearance of each video was computed based on the frontalized frames. Subsequently, for each pair of videos the ${\varDelta }\phi$ were extracted from the corresponding mean appearances and their dimensionality was reduced by applying PCA. Finally, a RBF-SVM classifier was used in order to predict the labels of the test pairs.

**Table 7:** YTF: Mean classification error and standard deviation

APEM-SIFT (Li et al. 2013)	$0.7854 \pm 0.0142$
STFRD+PMML (Cui et al. 2013)	$0.7948 \pm 0.0252$
DDML (Hu et al. 2014a)	$0.8126 \pm 0.0163$
LM3L (Hu et al. 2014b)	$0.8128 \pm 0.0117$
Eigen-PEP-SIFT (Li et al. 2014)	$0.8240 \pm 0.017$
APEM (fusion) (Li et al. 2013)	$0.7906 \pm 0.0151$
Eigen-PEP-SIFT (flip) (Li et al. 2014)	$0.8480 \pm 0.014$
RSF	$0.8051\pm 0.025$
RSF-V	$0.8320 \pm 0.015$

**Fig. 15:**
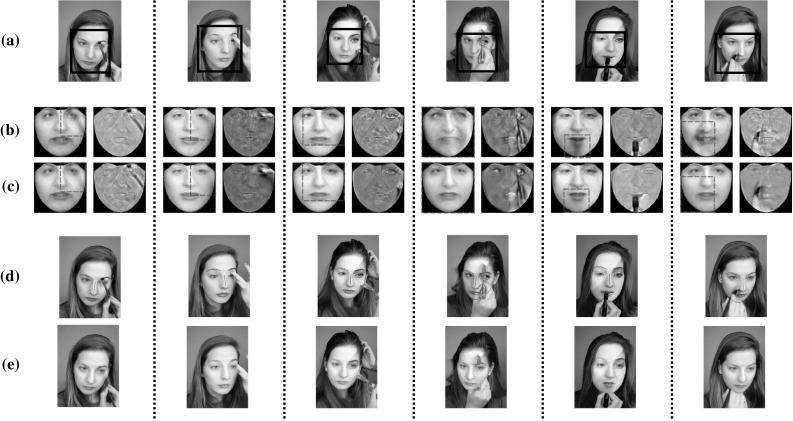
Video inpainting: **a** The detector (Zhu and Ramanan 2012) is used in order to locate the face in each frame. Then, the frontalized and error images for each frame are produced by employing **b** RSF or **c** RSF-V. By using **d** the landmark points obtained by RSF-V, the frontalized clean image is **e** back warped into input frame. As it can be observed, especially in the area defined by the *red boxes*, the quality of the frontalizations obtained by the RSF-V **c** are better than those produced by the RSF (**b**)

Given that the RSF-V was trained using only the provided images, we selected to compare its performance against that obtained by methods trained without flipped images. As shown in Table 7 the RSF-V outperforms all the compared methods that use only the provided images of the database. Please note that RSF-V achieves state-of-the-art results by employing only frontal images and IGOs features computed in one scale.

Futhermore, in order to show the effectiveness of the video-based RSF i.e, the V-RSF against to single frame RSF the following experiment has been conducted. We followed the same procedure like before and instead of producing the frontalized frames using the RSF-V, we applied the RSF independently in each frame. Then, the frontalized frames were used to compute the mean appearance in each video. Subsequently, the same feature extraction and classification steps were applied. The classification accuracy achieved by the frame-by-frame RSF is $0.8051 \pm 0.025$ while the accuracy of the RSF-V is $0.8320 \pm 0.015$. This improvement indicates that the incorporation of the temporal information in case of the RSF-V leads to frontalizations of better quality.

### Video Inpainting

The ultimate goal of video inpainting is to restore damaged areas or to remove unwanted elements from an image sequence. In order to investigate the effectiveness of the proposed method in this task, two image sequences were used: one from the movie 300 and another one depicting a woman during the make-up procedure (acquired from Youtube). The selected sequences are very challenging due to the presence of variations in poses, expressions, illumination conditions, image quality and occlusions. More specifically, occlusions due to hands, fingers, brushes, rings, and earrings are present in the videos. In addition the usage of different powders and creams had as result the change of the face appearance.

The aim of this experiment was to remove the unwanted elements from the faces in the whole sequence and produce a clean version of it. To this end, the position of the face in each frame was found by the detector in (Zhu and Ramanan 2012) and then the methods presented in Sects. 3 and 4 were employed in order to generate the clean frontal version of the face in each frame. Subsequently, the frontalized images were warped from the reference frame back to the original frame by using the corrected landmark points and the inverse warp function $\mathcal {W}^{-1}$. Figure  15 depicts results obtained for some representative frames of the test video. The frontalized and error images recovered from RSF and RSF-V are presented in Fig. 15b, c, respectively. As it can be observed (specifically inside the red dotted boxes), the results of RSF-V are of better quality which is attributed to information that all the faces of the subject span a low-rank subspace. By visually inspecting the results of inverse warping (Fig. 15d) it can be noticed that all occlusions have been removed and the recovered face is of a high quality. A video demonstrating the RSF-V is available at: https://www.youtube.com/watch?v=kSnFehb55O4&fmt=22 (When you watch the video please make sure you have enabled the full quality and resolution).

## Conclusions

In this paper, to the best our knowledge, we presented the first method that jointly performs landmark localization and face frontalization using only a simple statistical model based on few hundred frontal images. The proposed method outperforms state-of-the-art methods for face landmark localization that were trained on thousands of images in many poses and achieves comparable results in pose invariant face recognition and verification without using 3D elaborate models or features extracted by employing Deep-Learning methodologies.

## Footnotes

1 Some recent works based on deep learning shown that it may not be necessary to perform face frontalisation in order to achieve state-of-the-art performance (Schroff et al. 2015). Nevertheless, we believe that even in this cases face frontalisation is beneficial and could lead to even further performance improvement.

2 From experience we know that annotation of facial image with poses take in many cases twice the time compared with frontal poses (Sagonas et al. 2013b).

3 An open source implementation of the RSF is available at http://ibug.doc.ic.ac.uk/resources/rsf/.

4
https://github.com/jwyang/face-alignment, http://blog.dlib.net/, http://www.humansensing.cs.cmu.edu/intraface/.
